# Kaposi sarcoma presenting as small bowel obstruction

**DOI:** 10.1093/jscr/rjad385

**Published:** 2023-07-03

**Authors:** Jane Tian, Selma Janbey, Maryam Hassanesfahani, Shubham Bhatia, Martine A Louis, Noman Khan

**Affiliations:** Department of Surgery, Flushing Hospital Medical Center, Flushing, NY 11355, USA; New York Institute of Technology College of Osteopathic Medicine, Old Westbury, NY 11568, USA; Department of Surgery, Flushing Hospital Medical Center, Flushing, NY 11355, USA; Department of Surgery, Flushing Hospital Medical Center, Flushing, NY 11355, USA; Department of Surgery, Flushing Hospital Medical Center, Flushing, NY 11355, USA; Department of Surgery, Flushing Hospital Medical Center, Flushing, NY 11355, USA

**Keywords:** small bowel obstruction, Kaposi sarcoma, acquired immunodeficiency syndrome, human immunodeficiency virus, gastrointestinal tumors

## Abstract

Kaposi sarcoma (KS) is a low-grade tumor of the vascular endothelium. The majority of individuals affected have advanced human immunodeficiency virus (HIV) or acquired immunodeficiency syndrome (AIDS). The disease typically manifests as cutaneous lesions but reports have shown that systemic disease is not uncommon. Because gastrointestinal KS is often asymptomatic, it is likely underdiagnosed. Those with symptoms can present with vague abdominal pain, nausea/vomiting or anemia. Rarely the tumors can cause bowel obstruction or perforation. We present a case of small bowel obstruction cause by KS tumors in a young transgender male to female patient with poorly controlled AIDS, supported by literature review of the clinical presentation, diagnosis and treatment recommendations.

## INTRODUCTION

Kaposi sarcoma (KS) is the most common tumor seen in acquired immunodeficiency syndrome (AIDS) with typical findings of cutaneous lesions. Previous reports have shown that 10–66% of those patients with skin lesions also had systemic disease [1]. Although gastrointestinal involvement is not uncommon, it is often asymptomatic and as such, thought to be underdiagnosed. Usually, KS of the bowel is diagnosed as an incidental finding seen on imaging [2]. We present a case of KS presenting with obstruction and concern for bowel ischemia.

## CASE REPORT

**Figure 1:**
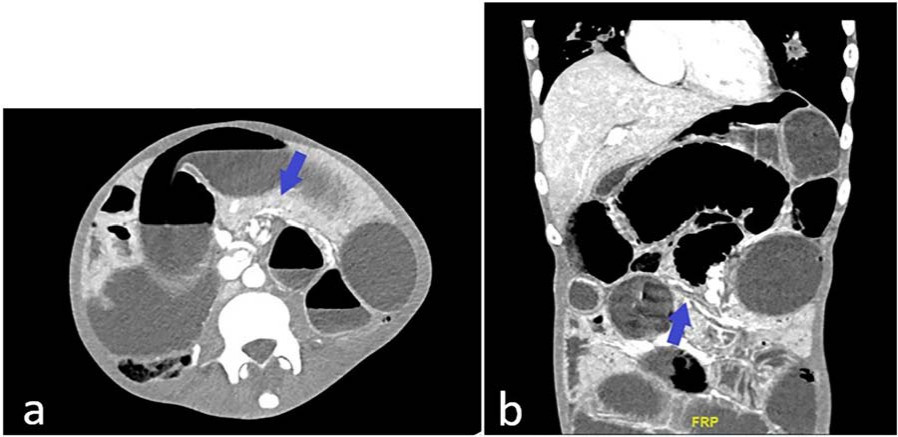
(**a**) CT abdomen and pelvis showing possible swirling of mesentery (blue arrow); (**b**) CT abdomen and pelvis showing dilated and collapsed small bowel loops suggestive of transition zone (blue arrow).

Patient is a frail 28-year-old transgender male to female with poorly controlled human immunodeficiency virus (HIV)/AIDS (CD4 count: 3, HIV viral load: 22000 copies/mL) who presented to the ED with one day of abdominal pain. History was limited as the patient was uncooperative. Vitals on arrival in the ED were: Temp 98°F, Pulse 78, BP 106/55, RR 18, SpO2 97%. The physical exam was significant for cachexia (BMI 17.3), skin changes compatible with skin type Kaposi’s Sarcoma, abdominal distention, and peritoneal signs. Computed tomography (CT) abdomen and pelvis showed multiple dilated small bowel loops with swirling of mesentery (Fig. 1a) and possible transition zone (Fig. 1b).

**Figure 2:**
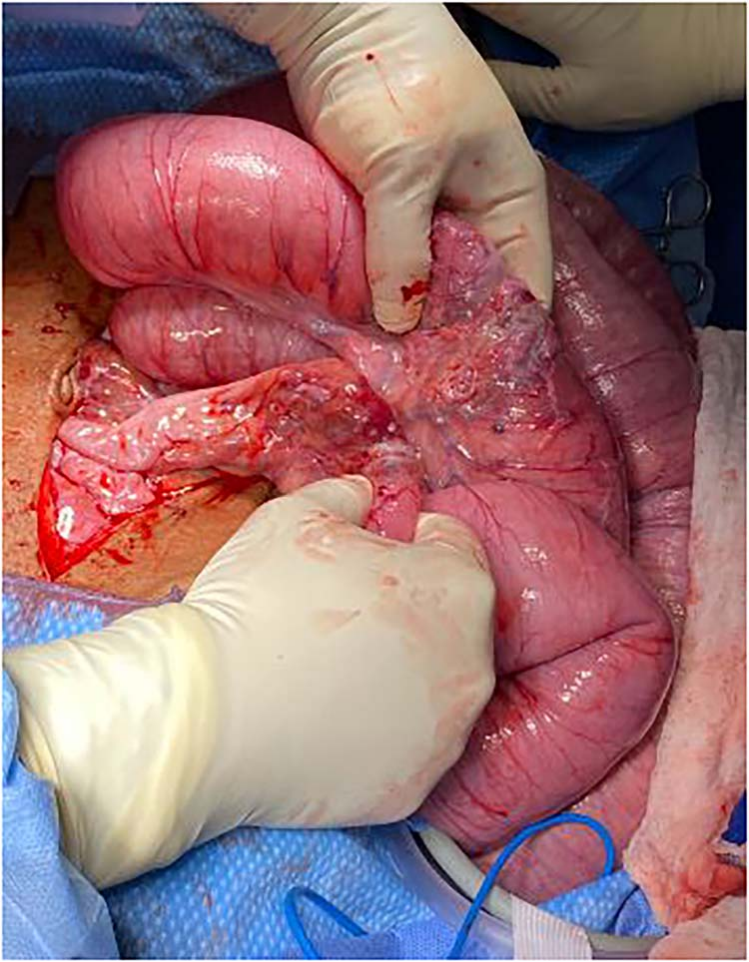
Intraoperative findings of dilated small bowel with transition zones from tumor.

**Figure 3:**
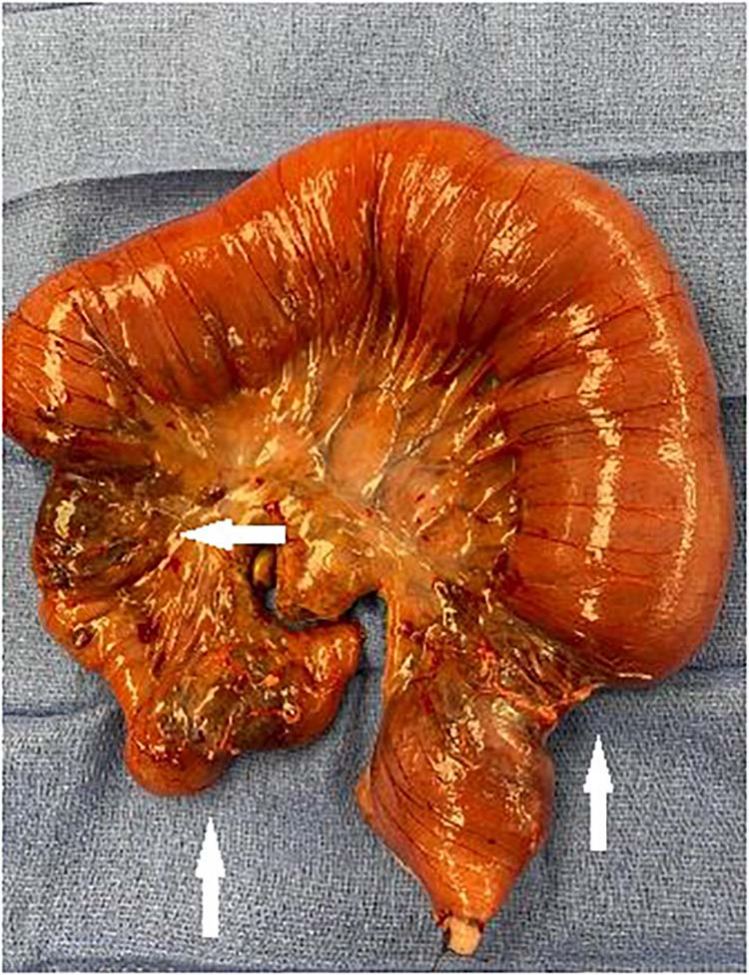
Segment of small bowel encompassing three tumors (white arrows) resected en bloc.

The patient was taken to the operating room for exploratory laparotomy given concerns for peritonitis in the context of closed loop obstruction. Intra-operative findings consisted of three ileal tumors spanning an approximately 40-cm segment, located 45 cm proximal to cecum accompanied by significant lymphadenopathy (Figs 2–3).

A palliative en bloc resection of the segment containing the three tumors was carried out with creation of an end ileostomy and mucous fistula. Final pathology showed malignant vascular tumor with extensive ulceration involving all layers of bowel wall, positive staining for CD-34 & HHV-8 consistent with KS.

The patient was started on HAART therapy post-operatively, had an uncomplicated hospital course and was eventually discharged to rehab. Unfortunately, the patient refused chemotherapy on discharge and was lost to follow up.

## DISCUSSION

KS is a vascular angio-proliferative neoplasm which usually presents on mucocutaneous sites but may involve visceral organs. In the US, KS predominantly affects HIV infected patients, with the majority of individuals having advanced immunosuppression (CD4 count <150 cells per cubic millimeter) and a high viral load (>10 000 copies per millimeter) at the time of diagnosis [1–3]. While gastrointestinal involvement is not uncommon, it is often asymptomatic and as such, thought to be underdiagnosed [4]. A greater than 50% incidence rate of KS of the GI tract has been seen in AIDs patients with cutaneous KS [5]. Symptoms of GI KS vary and may include abdominal pain, nausea, vomiting, diarrhea, or malabsorption. Rarely, advanced disease can present with gastrointestinal bleeding, intussusception, perforation, and, as in our patient, obstruction [6].

Given the indolent nature of GI KS, it raises the question of whether routine endoscopic screening should be done. Endoscopically KS appears as red or purple patchy lesions of different sizes and shapes of polypoid/nodular morphology. These lesions are typically identified in the stomach, esophagus, and duodenum [7]. The role of colonoscopy in screening remains controversial as previous case series demonstrated either low yield in identifying lesions or have seen concurrent upper and lower GI lesions [8]. Nagata et al. suggested obtaining routine endoscopy in all patients with cutaneous lesions, men who have sex with men and CD4+ T cell count <100/uL [9]. Of note, it has been reported that in New York, KS disproportionately affects the transgender population, with a reported proportional incidence ratio of 71.7 from 1979 to 2016 [10]. These rates underline the importance of public health screening and preventative care for this population.

In patients with AIDS-related KS with systemic manifestations, combined HAART and systemic chemotherapy is indicated. Liposomal doxorubicin is the agent of choice, and trials have shown that delivery via pegylated doxorubicin is effectively concentrated in lesions with greater tolerability and less toxicity than other chemotherapeutic agents [11–13].

The surgical management of small bowel obstruction due to KS remains controversial– the decision to perform surgery should be made on a case-by-case basis. In general, surgery may be indicated in cases of complete or high-grade partial obstruction, perforation, or ischemia, which are associated with a high risk of morbidity and mortality [14]. However, surgery may not always be feasible or appropriate in patients with advanced or disseminated KS who may have limited life expectancy or poor functional status [15]. In such cases, non-operative management such as supportive care or palliative measures may be more appropriate to relieve symptoms and maintain quality of life [16]. The decision to perform surgery should take into account various factors, including the patient’s overall health status, the extent and location of the obstruction, and the potential benefits and risks of surgery [17].

## CONCLUSION

KS should be included in the differential diagnosis of small bowel obstruction in patients with AIDS. Although this extent of progression of GI KS is rare, high index of suspicion and perhaps increased endoscopic screening is warranted when dealing with this vulnerable demographic.

## Data Availability

Data sharing is not applicable to this article as no new data were created or analyzed in this study.
